# Subtle cardiac dysfunction in lymphoma patients receiving low to moderate dose chemotherapy

**DOI:** 10.1038/s41598-021-86652-x

**Published:** 2021-03-29

**Authors:** Hsien-Yuan Chang, Chun-Hui Lee, Po-Lan Su, Sin-Syue Li, Ming-Yueh Chen, Ya-Ping Chen, Ya-Ting Hsu, Wei-Chuan Tsai, Ping-Yen Liu, Tsai-Yun Chen, Yen-Wen Liu

**Affiliations:** 1 Institute of Clinical Medicine, College of Medicine, National Cheng Kung University, Tainan, Taiwan; 2 Division of Cardiology, Department of Internal Medicine, National Cheng Kung University Hospital, College of Medicine, National Cheng Kung University, Tainan, Taiwan; 3 Division of Hematology and Oncology, Department of Internal Medicine, National Cheng Kung University Hospital, College of Medicine, National Cheng Kung University, Tainan, Taiwan; 4 Division of Chest, Department of Internal Medicine, National Cheng Kung University Hospital, College of Medicine, National Cheng Kung University, Tainan, Taiwan

**Keywords:** Lymphoma, Cardiology

## Abstract

Left ventricular (LV) global peak systolic longitudinal strain (GLS) is a sensitive measurement for detecting subtle LV systolic dysfunction and a powerful prognostic predictor. However, the clinical implication of LV GLS in lymphoma patients receiving cancer therapy remains unknown. We prospectively enrolled 74 lymphoma patients (57.9 ± 17.0 years old, 57% male). We performed echocardiographic studies after the 3rd and 6th cycles and 1 year after chemotherapy and a cardiopulmonary exercise test upon completion of 3 cycles of anticancer therapy. Cancer therapy-related cardiac dysfunction (CTRCD) was defined as a ≥ 15% relative reduction in GLS value from baseline. The primary outcome was a composite of all-cause mortality and heart failure events. Thirty-six patients (49%) had CTRCD (LV GLS: baseline vs. after 3rd cycle of therapy: 20.1 ± 2.6 vs. 17.5 ± 2.3%, *p* < 0.001). CTRCD was detected after the 3rd cycle of anticancer therapy. CTRCD patients had impaired exercise capacity (minute oxygen consumption/kg, CTRCD vs. CTRCD (-): 13.9 ± 3.1 vs. 17.0 ± 3.9 ml/kg/min, *p* = 0.02). More primary outcome events occurred in the CTRCD group (hazard ratio 3.21; 95% confidence interval 1.04–9.97; *p* = 0.03). LV GLS could detect subtle but clinically significant cardiac dysfunction in lymphoma patients in the early stage of anticancer therapy. CTRCD may be associated with not only a reduced exercise capacity but also a worse prognosis.

## Introduction

Cardiovascular diseases and neoplasms are the leading causes of death worldwide^1,2^. In the last decade, enormous advances in anti-neoplastic therapeutics have led to a marked reduction in cancer mortality, but an increasing number of unintended cardiovascular consequences have been reported in cancer survivors^3^. These cancer therapy-related cardiac dysfunctions (CTRCDs) include heart failure, conduction disorders, hypertension, thromboembolic events and ischemia^4–6^. Heart failure is one of the most notorious cancer therapy-related cardiotoxicities and significantly impacts the prognosis of cancer survivors^7,8^.

Traditional 2-dimensional echocardiography could only identify notably impaired left ventricular (LV) systolic contractility but failed to detect the early deterioration of LV systolic function. Compared to conventional echocardiographic parameters, such as LV ejection fraction, speckle-tracking echocardiography (STE) with myocardial deformation analysis, such as LV global peak systolic longitudinal strain (GLS), has been indicated to be a feasible, objective and more sensitive modality to detect subtle but clinically significant LV systolic dysfunction and is a powerful prognostic predictor^9–14^. It was reported that LV GLS could be used to identify CTRCD in breast cancer patients after they completed cancer therapy^15–19^. Although it is recommended to use LV GLS to assess heart function prior to, during, and after chemotherapy^18–20^, how to define subtle LV dysfunction according to LV GLS is not well clarified, and the prognostic impact of subtle LV systolic dysfunction in cancer survivors is not clearly illustrated. Moreover, the cardiopulmonary exercise test (CPET) is an important clinical modality for assessing integrative exercise responses involving the pulmonary, cardiovascular, hematopoietic, neuropsychological, and skeletal muscle systems^21,22^. Nevertheless, there are limited studies using CPET to investigate cardiotoxicity in lymphoma patients receiving standard chemotherapy. Therefore, we conducted this prospective cohort study to investigate the clinical application of LV GLS in evaluating the early impact of cancer therapy on cardiac function and the clinical implication of subtle LV dysfunction in lymphoma patients during cancer therapy.

## Methods

We prospectively enrolled patients with newly diagnosed lymphoma (age ≥ 20 years) at the National Cheng Kung University Hospital between April 2017 and March 2019. All patients received anthracycline-based anticancer therapy. The exclusion criteria were moderate or severe aortic or mitral valve disease, pregnancy, breastfeeding, a prior history of chemotherapy or radiotherapy, atrial fibrillation with very irregular ventricular response, poor image quality in the GLS analysis, and unwillingness to participate in the study. This study adhered to the Declaration of Helsinki, and study approval was obtained from the Human Research and Ethics Committee of the National Cheng Kung University Hospital (IRB numbers: A-ER-105-407 and B-ER-106-392). All enrolled patients provided written informed consent. The enrolled patients’ medical records during the follow-up period (February 2017 to December 2019) were carefully reviewed. Clinical data on age, sex, comorbidities, medical history, cancer pathology, cancer stage, concomitant chemotherapy comorbidities, medical history, and anticancer regimen were obtained through medical record review. None of the patients received other cardiotoxic agents or radiation therapy or underwent cardiac protective protocols during the procedure.

### Outcomes

The primary outcome was defined as a composite of all-cause mortality or worsening heart failure events. Heart failure events included pulmonary edema on chest X-ray, hospitalization for heart failure, or an emergency room visit for intravenous therapy of loop diuretics for heart failure.

The secondary outcomes included the Common Terminology Criteria for Adverse Events Version 5 (CTCAE V5) score for heart failure evaluated at the following time points: after the 3rd and 6th cycle and 1 year after the initiation of anticancer therapy.

### Echocardiography

All subjects underwent echocardiography prior to chemotherapy; after the 1st, 3rd, and 6th cycle; and 1 year later. Patients were examined in the left lateral decubitus position by well-trained echocardiographers using an ultrasound system with a 2–5 MHz probe (Vivid-E9, GE Healthcare, Horten, Norway). Based on the recommendations of the American Society of Echocardiography, quantifications of LV mass index, LV EF, and left atrium volume index (LAVi) were performed^23^. LAVi was calculated as (A1 x A2/L) × 8/3π, where L is the average LA length in the apical four- and two-chamber views. Mitral inflow velocity measurements included early (E) and late (A) peak mitral inflow velocities and the E/A ratio. We acquired pulsed tissue Doppler imaging from the apical 4-chamber view and placed the sample volume on the LV septal and the lateral mitral annulus to obtain peak early diastolic velocity (e′). The ratio of early mitral inflow velocity to early diastolic mitral annular velocity (E/e′) was calculated from the average of the septal and lateral e′ (average E/e′ = E/[(e_septal_′ + e_lateral_′)/2]). All images were acquired in 3 consecutive cardiac cycles and stored digitally with a frame rate of 50–90 frames/s for subsequent offline analysis.

### Myocardial deformation (strain) analysis

Two cardiologists who were blinded to the clinical information used automated function imaging (AFI) software (EchoPAC workstation, BT11, GE-VingMed, Horten, Norway) to measure LV GLS from three standard apical views. Each apical view assessment produced six segmental values of peak systolic longitudinal strain. Thus, LV GLS was defined as the mean of the peak systolic longitudinal strain of all the LV segments from three apical views. Additionally, analysis of the different myocardial layers was performed offline using AFI software. For each apical view, the AFI software automatically separated the myocardium into the subendocardial and subepicardial layers. According to the European Society of Cardiology guidelines^19^, CTRCD was defined as a relative reduction in GLS from baseline ≥15%.

### Cardiopulmonary exercise test

The enrolled patients underwent CPET using a cycle ergometer (MasterScreen CPX, CareFusion, CA, USA) in an upright position with a standardized protocol. Patients with a poor performance status or musculoskeletal disorder were excluded from undergoing CPET^24^. We recorded the minute oxygen consumption (VO_2_), carbon dioxide production (VCO_2_), minute ventilation (VE), end-tidal carbon dioxide (P_ET_CO_2_), and heart rate. The heart rate reserve (HRR), for which less than 15 is defined as low, was the difference between the maximal heart rate during CPET and the subject’s maximal predicted value. The ventilatory reserve, for which greater than 85% is defined as low, was the ratio of the maximal minute ventilation during CPET to the maximal voluntary ventilation. The oxygen pulse (VO_2_/HR), VE/VO_2_, VE/VCO_2_, and respiratory quotient (VCO_2_/VO_2_) were averaged every 10 seconds. The peak oxygen consumption (peak VO_2_) was determined as the highest value of the 30-second average value of oxygen consumption. The anaerobic threshold was defined using the V-slope method^25^. All eligible patients underwent CPET upon completing 3 cycles of anticancer therapy.

### Statistical analysis

IBM SPSS Statistics V21.0 software (IBM Corp. Released 2012. IBM SPSS Statistics for Windows, Version 21.0. Armonk, NY: IBM Corp.) was used for statistical analysis. Continuous data are presented as the mean ± standard deviation or as the median (interquartile range), depending on the distribution. Dichotomous data are presented as numbers and percentages. Comparisons were conducted using Student’s *t*-test or the Mann-Whitney U test for continuous variables that showed a normal or nonparametric distribution, respectively. Chi-square test or Fisher’s exact test was used for categorical variables where appropriate. Using the Bland-Altman analysis of agreement^26^ and the interclass correlation coefficient, 30 randomly selected patients’ LV GLS measurements were applied to assess the intra- and inter-rater reliability. GLS was independently measured by two independent observers. For intra-rater variability, we repeated the same measurement 1 month apart. A paired *t*-test was used to determine the significance between EF and the change in GLS according to time. The Kaplan-Meier method was used with a log-rank test to compare event-free rates between strata. A univariate Cox regression analysis was performed to evaluate factors associated with the primary endpoint. Factors with a value of *p* < 0.1 based on the univariate Cox regression analysis were included in the multivariate Cox regression analysis to identify factors independently associated with anticancer therapy-related subtle LV dysfunction.

## Results

### Baseline demographic characteristics (Table 1)

**Table 1:** Baseline demographic characteristics of the lymphoma patients post anti-cancer therapy with and without left ventricular dysfunction.

	Total (N = 74)	CTRCD (N = 36)	Non-CTRCD (N = 38)	*p*
Age (years old)	57.9 ± 17.0	59.6 ± 17.5	56.3 ± 16.6	0.42
Male	42 (57%)	26 (72%)	16 (42%)	0.01
**Lymphoma subtype**				
Hodgkin lymphoma	5 (7%)	1 (3%)	4 (11%)	0.87
T cell lymphoma	8 (11%)	5(17%)	3 (8%)	
B cell lymphoma (non-DLBCL)	26 (35%)	13 (36%)	13 (34%)	
DLBCL	35 (47%)	17 (47%)	18 (47%)	
**Therapy regimens**				
R-CHOP21	28 (38%)	13 (36%)	15 (40%)	0.82
RCOP	12 (16%)	8 (22%)	4 (11%)	
DA-EPOCH-R	10 (14%)	4 (11%)	6 (16%)	
R-mini-CHOP	6 (8%)	1 (3%)	5 (13%)	
CHOEP	4 (5%)	2 (6%)	2 (5%)	
ABVD	5 (7%)	1 (3%)	4 (11%)	
Others	9 (12%)	7 (19%)	2 (5%)	
**Comorbidities**				
Hypertension	22 (30%)	12 (33%)	10 (26%)	0.51
Diabetes mellitus	10 (14%)	6 (17%)	4 (11%)	0.51
Coronary artery disease	3 (4%)	2 (6%)	1 (3%)	0.24
Heart failure	0	0	0	0.99
Hemoglobin < 11 g/dL	18 (24%)	13 (36%)	5 (13%)	0.02
**Laboratory data**				
White blood cell count (10^3^/µL)	9.9 ± 14.3	8.5 ± 3.2	11.2 ± 19.8	0.42
Platelet count (10^3^/µL)	286.9 ± 139.0	286.7 ± 123.4	287.2 ± 154.0	0.99
LDH level (U/L)	330.1 ± 264.6	284.6 ± 112.9	372.1 ± 347.5	0.15
Creatinine (mg/dL)	0.9 ± 0.9	1.1 ± 1.3	0.8 ± 0.2	0.12
Albumin (mg/dL)	4.1 ± 0.6	4.1 ± 0.6	4.2 ± 0.6	0.55
Calcium (mg/dL)	9.2 ± 0.7	9.2 ± 0.8	9.2 ± 0.5	0.73
Uric acid (mg/dL)	5.9 ± 2.1	5.9 ± 2.2	5.8 ± 2.0	0.84
β2 microblobulin (mg/L)	3.8 ± 6.0	5.3 ± 8.8	2.6 ± 1.4	0.18
IgG (mg/dL)	1470.7 ± 1232.5	1621.1 ± 1600.6	1276.1 ± 417.2	0.39
IgA (mg/dL)	232.5 ± 119.1	213.5 ± 124.8	257.2 ± 109.9	0.26
IgM (mg/dL)	179.0 ± 613.8	247.3 ± 817.9	90.7 ± 46.8	0.44
HBsAg (+)	12 (16%)	5 (14%)	7 (18%)	0.60
HCV Ab (+)	5 (7%)	1 (3%)	4 (11%)	0.36
**Cumulative dose of anti-cancer drugs**				
Doxorubicin (mg/m^2^)	201.4 ± 92.5	221.4 ± 90.1	186.3 ± 92.7	0.15
Rituximab (mg/m^2^)	2212.3 ± 556.5	2256.2 ± 727.0	2165.1 ± 285.5	0.54
Cyclophosphamide (mg/m^2^)	4010.0 ± 1286.8	3986.1 ± 1118.2	4033.9 ± 1453.3	0.88
Vincristine (mg/m^2^)	5.5 ± 2.4	6.0 ± 2.3	5.0 ± 2.4	0.11
Etoposide(mg/m^2^)	337.0 ± 156.5	374.6 ± 162.6	303.2 ± 151.0	0.34

Chemotherapy regimens: R-CHOP 21: Rituximab, cyclophosphamide, doxorubicin, vincristine and prednisolone; administered every 21 days. R-COP: Rituximab, cyclophosphamide, vincristine and prednisolone. DA-EROCH-R: dose-adjusted etoposide, prednisone, vincristine, cyclophosphamide, doxorubicin, and rituximab. R-mini-CHOP: Rituximab, low dose of cyclophosphamide, vincristine and prednisolone. CHOEP: cyclophosphamide, doxorubicin, vincristine, etoposide and prednisolone. ABVD: Doxorubicin, bleomycin, vinblastine and dacarbazine. Data are expressed as mean ± SD or number (%).

CTRCD indicates cancer therapy-related cardiac dysfunction; DLBCL, diffused large B cell lymphoma; HBsAg, hepatitis B surface antigen; HCV Ab, hepatitis C viral antibody; Ig, immunoglobulin; LDH, lactate de and LV, left ventricular.

Ninety-one patients with lymphoma receiving anticancer therapy were prospectively screened. Owing to the following reasons, 17 patients were excluded: radiotherapy (n = 1), inadequate image to analyze (n = 3), loss to follow-up (n = 3), follow-up refusal (n = 4), and death after the 1st cycle of chemotherapy (n = 6). Seventy-four patients (57.9 ± 17.0 years old, 57% male) who completed all the echocardiographic studies, i.e., at baseline (prior to anticancer therapy), after the 3rd and 6th cycles of anticancer therapy, and 1 year after anticancer therapy, were included in the final analyses. According to the change in LV GLS, the patients were stratified into two groups: the CTRCD group (a relative reduction in GLS value from baseline ≥ 15%; n = 36, age 59.6 ± 17.5 years) and the non-CTRCD group (a relative reduction in GLS value from baseline < 15%; n = 38, age 56.3 ± 16.6 years). There was no significant difference in baseline demographic characteristics between these two groups, except for male sex and anemia (hemoglobin (Hb) < 11 g/dl). The CTRCD group included more male patients and had a higher rate of anemia (Table 1).

### Evaluation of cardiac function (Table 2)

**Table 2:** Longitudinal follow-up results of echocardiographic studies.

Parameters	Total (N = 74)	p*	CTRCD (N = 36)	p*	Non-CTRCD (N = 38)	p*	*p*#
**LA volume index (mL/m**^**2**^**)**							
Baseline	26.7 ± 9.0		27.4 ± 8.8		26.1 ± 9.3		0.52
Post-treatment 3 cycles	28.8 ± 10.5	0.44	27.8 ± 11.4	0.82	28.1 ± 9.8	0.38	0.90
Post-treatment 6 cycles	27.6 ± 9.8	0.69	26.4 ± 7.8	0.46	28.8 ± 11.6	0.05	0.34
Post-treatment 1 year	28.3 ± 11.0	0.89	26.0 ± 7.7	0.30	31.5 ± 13.8	0.31	0.12
**LV ESVi (mL/m**^**2**^**)**							
Baseline	19.2 ± 7.0		19.2 ± 7.5		19.2 ± 6.6		1.00
Post-treatment 3 cycles	19.1 ± 9.0	0.85	19.7 ± 11.1	0.64	18.5 ± 6.5	0.32	0.55
Post-treatment 6 cycles	18.4 ± 7.2	0.53	19.0 ± 8.0	0.69	17.8 ± 6.5	0.16	0.49
Post-treatment 1 year	19.7 ± 7.5	0.58	19.8 ± 6.9	0.60	19.5 ± 8.3	0.82	0.89
**LV EDVi (mL/m**^**2**^**)**							
Baseline	61.4 ± 14.6		62.9 ± 15.1		60.1 ± 14.2		0.40
Post-treatment 3 cycles	62.9 ± 15.7	0.34	62.4 ± 17.1	0.80	63.4 ± 14.4	0.12	0.77
Post-treatment 6 cycles	59.8 ± 14.5	0.60	60.2 ± 13.4	0.45	59.4 ± 15.6	0.94	0.83
Post-treatment 1 year	61.5 ± 14.8	0.69	60.3 ± 13.1	0.42	63.2 ± 16.9	0.80	0.52
**LVEF (%)**							
Baseline	69.1 ± 7.0		70.2 ± 5.7		67.8 ± 7.9		0.17
Post-treatment 3 cycles	70.1 ± 8.8	0.26	69.3 ± 10.2	0.57	71.0 ± 7.3	< 0.001	0.41
Post-treatment 6 cycles	69.5 ± 7.3	0.58	68.6 ± 8.4	0.27	70.5 ± 5.9	0.03	0.31
Post-treatment 1 year	68.5 ± 6.9	0.35	67.7 ± 6.8	0.26	69.7 ± 6.9	0.99	0.33
**E/A**							
Baseline	1.0 ± 0.4		0.9 ± 0.3		1.1 ± 0.5		0.13
Post-treatment 3 cycles	1.0 ± 0.4	0.73	1.0 ± 0.4	0.44	1.0 ± 0.4	0.21	0.50
Post-treatment 6 cycles	0.9 ± 0.4	0.02	0.9 ± 0.3	0.12	1.0 ± 0.4	0.09	0.09
Post-treatment 1 year	1.1 ± 0.6	0.96	0.9 ± 0.4	0.99	1.2 ± 0.8	0.95	0.12
**Average E/e′**							
Baseline	8.0 ± 3.0		8.4 ± 2.7		7.7 ± 3.2		0.35
Post-treatment 3 cycles	8.5 ± 4.1	0.26	8.9 ± 4.4	0.46	8.1 ± 3.8	0.30	0.38
Post-treatment 6 cycles	8.5 ± 3.9	0.21	8.9 ± 3.8	0.33	8.1 ± 4.0	0.44	0.42
Post-treatment 1 year	7.8 ± 3.2	0.11	7.8 ± 2.1	0.35	7.8 ± 4.2	0.16	0.99
**Absolute value of LV GLS (%)**							
Baseline	19.7 ± 2.5		20.1 ± 2.6		19.3 ± 2.2		0.16
Post-treatment 3 cycles	18.4 ± 2.6	< 0.001	17.5 ± 2.3	< 0.001	19.2 ± 2.6	0.68	0.01
Post-treatment 6 cycles	17.6 ± 3.0	< 0.001	16.3 ± 2.2	< 0.001	19.6 ± 2.7	0.87	< 0.001
Post-treatment 1 year	18.3 ± 2.4	< 0.001	18.2 ± 2.3	< 0.001	18.5 ± 2.5	0.05	0.70

Data are expressed as mean ± SD or number (%).

CTRCD indicates cancer therapy-related cardiac dysfunction; EDVi, end-diastolic volume index; EF, ejection fraction; ESVi, end-systolic volume index; GLS, global peak systolic longitudinal strain; LA, left atrial; LV, left ventricular.

p*: comparison between the tested value and the baseline value.

*p*#: comparison between the subtle LV dysfunction group and the non-subtle LV dysfunction group.

**Figure 1:**
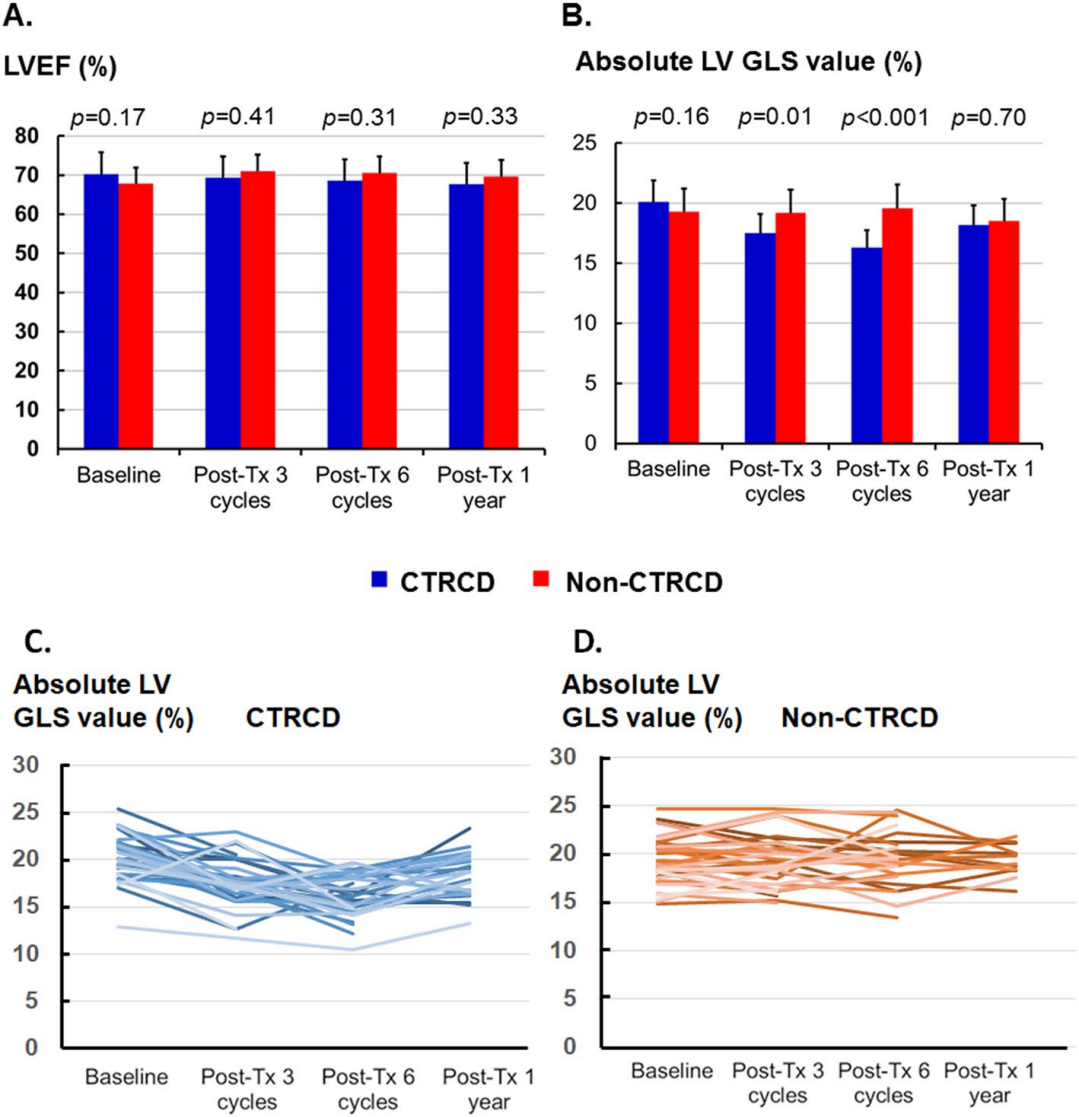
Time trend of LV EF and GLS. (**A**) There is no change in EF. (**B**) In patients with subtle LV dysfunction, the absolute GLS value was significantly decreased after 3 cycles (*p* = 0.01) and 6 cycles of (*p* = 0.001) anticancer therapy and partially recovered 1 year later. (**C**,**D**) Line graph of absolute GLS value (%) changes in patients with CTRCD (**C**) and without CTRCD (**D**).

Compared to the baseline echocardiographic study, there was no significant difference in LV geometry (i.e., LV end-diastolic volume index and LV end-systolic volume index), EF, or diastolic function (including LAVi, E/A, and average E/e′) after the 3rd and 6th cycles and 1 year after anticancer therapy (Table 2, Fig. 1A). Only one patient had a reduced LV EF (< 50%) during the treatment course. Furthermore, LV GLS was not significantly lower after the 1st cycle (Supplemental Figure 1), but the absolute LV GLS value was significantly lower after the 3rd cycle of anticancer therapy (Fig. 1B, Table 2), which indicated subclinical LV systolic dysfunction.

Compared to the non-CTRCD group, the CTRCD group had neither LV remodeling nor LV diastolic dysfunction, but a lower absolute LV GLS value (Fig. 1C,D. CTRCD group vs. non-CTRCD group: 17.5 ± 2.3% vs. 19.2 ± 2.6%, *p *= 0.01, post-3rd cycle treatment; 16.3 ± 2.2% vs. 19.6 ± 2.7%, *p* < 0.001, post-6th cycle treatment) was noted in the CTRCD group despite their LV EF value not being decreased (Table 2). Importantly, the cumulative dose of either anthracycline or other anticancer regimens was not different between the groups (Table 1). After adjusting for male sex and anemia (Hb < 11 g/dl), neither age nor cumulative doxorubicin dose was independent risk factors for CTRCD (Supplemental Table 2).

### CPET results in the groups with and without CTRCD (Table 3)

**Table 3:** Cardiopulmonary exercise test (CPET) results between the subtle left ventricular (LV) dysfunction group and the non-subtle LV dysfunction group.

CPET parameters	Subtle LV dysfunction (n = 12)	No Subtle LV dysfunction (n = 36)	*p*
mVO_2_ (ml/kg/min)	13.9 ± 3.1	17.0 ± 3.9	0.02
mVO_2_ AT (ml/kg/min)	7.0 ± 1.5	9.8 ± 3.3	0.02
Low heart rate reserve	5 (42%)	18 (50%)	0.62
VE reserve limitation	2 (17%)	10 (28%)	0.7
VE/VCO_2_ AT	41.6 ± 7.5	37.1 ± 6.8	0.08

VE reserve limitation: VE > 0.85 maximum VE. Low heart rate reserve: 220 – age – Heart rate > 15. Data are expressed as mean ± SD.

AT, at anaerobic threshold; mVO_2,_ maximum oxygen uptake; VE, minute ventilation.

Because the patients had poor general physical status at enrollment and could not tolerate undergoing CPET, we did not conduct baseline CPET. However, after the 3rd cycle of anticancer therapy, only 48 patients were well enough to undergo CPET. Among these 48 patients, CTRCD patients had lower VO_2_/kg (13.9 ± 3.1 vs. 17.0 ± 3.9 ml/kg/min, *p* = 0.02) and lower VO_2_/kg at the anaerobic threshold (7.0 ± 1.5 vs. 9.8 ± 3.3 ml/kg/min, *p* = 0.02, Table 3), indicating a reduced exercise capacity due to cardiac systolic dysfunction. Thus, this study showed that CPET confirmed the diagnostic definition of CTRCD in terms of cardiac systolic dysfunction.

### Outcomes

**Figure 2:**
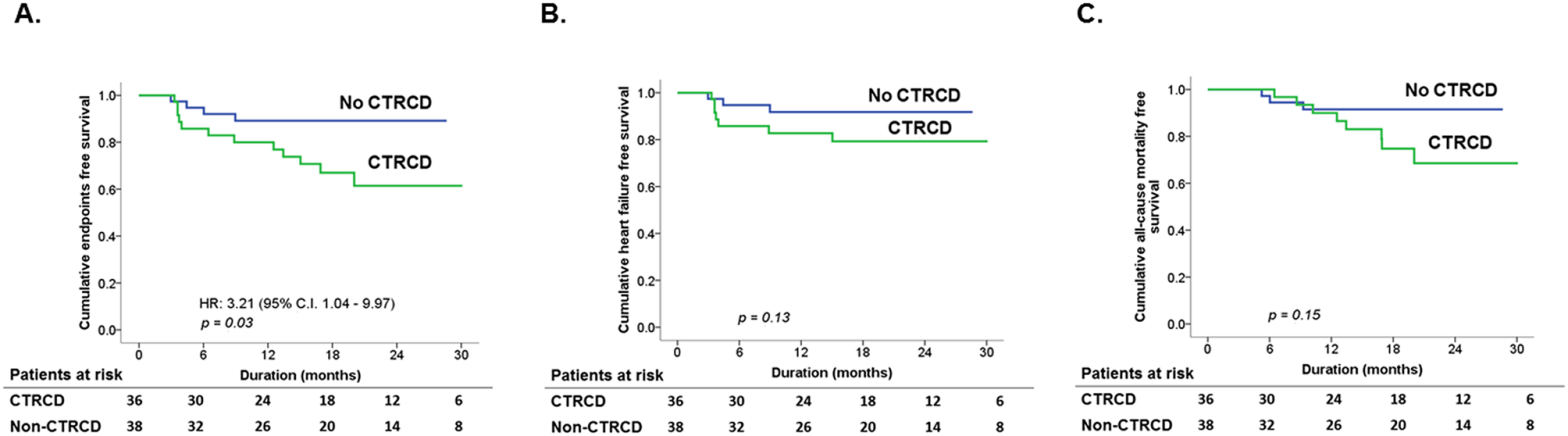
Kaplan–Meier curve of cumulative survival. (**A**) Compared to patients without cancer therapy-related cardiac dysfunction (CTRCD), patients with CTRCD had a significantly higher incidence of the primary endpoint (defined as a composite of all-cause mortality or worsening heart failure events) (*p* = 0.03). (**B**,**C**) There was no significant difference in heart failure events or all-cause mortality between patients with and without CTRCD (*p* = 0.13 and 0.15, respectively).

The mean follow-up duration was 1.4 ± 0.6 years, and 16 (22%) patients reached end points. The incidence of the primary composite outcome was higher in the CTRCD group than in the non-CTRCD group (hazard ratio 3.21; 95% CI 1.04 to 9.97; *p* = 0.03, Table 4 and Fig. 2A). After adjusting for sex and anemia, CTRCD was an independent predictor of the primary endpoint. However, the CTRCD group appeared to have a higher incidence of either heart failure or all-cause mortality, but the difference between the groups was not significant (Table 4, Fig. 2B,C). Moreover, there was no difference in the CTCAE heart failure score between both groups after the 3rd and 6th cycles and 1 year after chemotherapy (Table 4).

**Table 4:** Primary and secondary outcomes between the cancer therapy-related cardiac dysfunction (CTRCD) group and the non-CTRCD group.

	CTRCD (N = 36)	Non-CTRCD (N = 38)	*p*
Primary endpoints (i.e. all-cause mortality and heart failure)	12 (33%)	4 (11%)	0.03
All-cause mortality	8 (22%)	3 (8%)	0.15
Heart failure	7 (19%)	3 (8%)	0.13
**Secondary endpoints**			
CTCAE HF score			
Post-treatment 3 cycles	0.0 ± 0.2	0.1 ± 0.4	0.44
Post-treatment 6 cycles	0.4 ± 0.8	0.3 ± 0.7	0.43
Post-treatment 1 year	0.3 ± 0.7	0.5 ± 1.0	0.47

Data are expressed as mean ± SD or number (%).

CTCAE HF, Common Terminology Criteria for Adverse Events heart failure; LV, left ventricular.

### Inter- and intra-rater variability

The intra- and interrater correlation coefficients of the average measures for GLS were 0.893 (95% CI 0.775–0.949) and 0.925 (95% CI 0.843–0.965), respectively. The mean intra- and interrater differences [mean ± standard deviation (95% limits of agreement)] for GLS were 0.16±1.13 (− 2.05 to 2.37) and − 0.45±1.20 (− 2.80 to 1.90), respectively. The Bland-Altman analysis demonstrated no systemic bias in LV GLS between intra- and interrater agreements (Supplemental Figure 2).

## Discussion

This is the first study to demonstrate that the incidence of CTRCD was up to 49% despite low cumulative doses of anticancer therapy. Importantly, this is the first study to confirm that lymphoma patients with subtle LV dysfunction had impaired exercise capacity, as shown by CPET. Notably, the CTRCD patients had subtle LV systolic dysfunction and a higher risk of all-cause mortality and heart failure events, although the CTCAE heart failure score did not show a difference between the CTRCD and non-CTRCD patients.

In this study, LV GLS derived from STE was shown to be a verified and feasible noninvasive technique for the early detection of subclinical LV dysfunction in lymphoma patients receiving cancer therapy. Our result is consistent with that of a previous study in that GLS decreased with the number of chemotherapy cycles and the GLS value increased slightly after discontinuing chemotherapy for six months^27^. The low cumulative dose of cancer therapy regimens in our patients could lead to a lack of significant changes in conventional echocardiographic parameters, such as LV EF, LAVi, average E/e′, etc. It is worth mentioning that LV GLS could identify patients with CTRCD who had a reduced exercise capacity resulting from subtle LV dysfunction, which was confirmed by CPET. This finding demonstrated the correlation between patients’ objective function and CTRCD^27^.

Mounting evidence has shown that LV GLS is a very powerful prognostic predictor, not only for patients with cardiac disease but also for those with systemic diseases, such as hypertension^12^, septic shock^14^, and chronic kidney disease^11,13^. Here, we demonstrated the prognostic value of LV GLS in patients with lymphoma undergoing cancer therapy. Patients with subtle LV dysfunction had a significantly increased risk of major adverse events (i.e., all-cause mortality and heart failure events, HR 3.21, 95% CI 1.04–9.97). It is worth noting that only one patient had LV dysfunction, which was defined as EF < 50%. Although most patients with CRTCD had only subtle LV dysfunction but not EF less than 50%, CTRCD truly affected the patients’ prognosis. Therefore, more research is warranted to determine how to prevent CTRCD and improve cardiac function in CTRCD patients.

Female sex was recognized to be associated with the risk of cardiotoxicity following anthracycline treatment^19^. However, many studies have shown that male sex is a predictor of LV dysfunction after doxorubicin therapy as well as a risk factor for subclinical late cardiomyopathy in adult lymphoma patients receiving doxorubicin^28,29^. Moreover, men had a greater incidence of major adverse cardiac events than women after anthracycline therapy^30^. Additionally, the prevalence of anemia is higher in lymphoma patients and is considered an adverse prognostic factor for overall survival and progression-free survival. Furthermore, a retrospective analysis that examined anticancer therapy-associated heart failure demonstrated that anemic patients have a higher risk of LV dysfunction^31^.

CPET can provide a thorough assessment of integrative exercise physiology involving the pulmonary, cardiovascular, muscular, and cellular oxidative systems. Furthermore, CPET plays an important role in cardiology in terms of including several forms of exercise intolerance, with a predominant focus on heart failure with reduced or preserved EF^21^. In our cohort, patients with subtle LV dysfunction had lower VO2 and VO2 at the anaerobic threshold according to the CPET results. Despite physical exhaustion, CPET can also be considered an adjuvant tool to detect subclinical cardiotoxicity in conjunction with STE. However, some patients with a poor performance status or musculoskeletal disorder refuse to undergo CPET because of the associated discomfort. Finally, only 61% of patients had received CPET in our study. Thus, echocardiography with GLS may be a better and more comfortable tool than CPET for screening chemotherapy-related cardiotoxicity. However, investigation assessment scores, such as the CTCAE heart failure score, did not reveal a significant difference in either group, suggesting that the traditional clinical score is not sensitive enough to detect chemotherapy-related subtle LV dysfunction.

The factors that influence the risk of developing cancer therapeutic-related cardiac dysfunction may be subdivided into three groups: patient-related clinical risk factors, treatment-related risk factors, and the individual genetic profile^32^. Cancer and cardiovascular disease share some risk factors, such as tobacco smoking, unhealthy diet, obesity, chronic inflammation, and increased oxidative stress. Moreover, exposure to radiation from diagnostic assessments, epigenesis, and regenerative signaling also have potential associations with both illnesses^7,33^. Notably, there is interpatient variability despite the established clinical and cardiovascular risk factors for anthracycline-related cardiotoxicity. Several studies disclosed cardiotoxicity-related pharmacogenetic variants after anthracycline treatment. The genotypes *CBR3*, *CELF4*, and *HAS3* in pediatric cancer patients are associated with the dose-response relationship between anthracycline exposure and the risk of cardiomyopathy^34^. The results from the meta-analyses revealed that *ABCC2 rs8187710*, *CYBA rs4673*, and *RAC2 rs13058338* genetic polymorphisms played an important role in anthracycline-induced cardiotoxicity^35^. The current evidence regarding the molecular foundations of early or delayed anticancer therapy-related cardiotoxicity remains unclear.

## Study limitations

There are some limitations to this study. First, the sample size is small; however, this is the first prospective cohort study to demonstrate that subtle cancer therapy-related LV dysfunction in lymphoma patients detected by GLS has prognostic value. Second, 39% of patients did not undergo CPET. Third, the chemotherapy regimens varied. Thus, the correlation between subtle LV dysfunction and drug combination is uncertain. Finally, we did not measure the levels of cardiac biomarkers, such as high-sensitivity cardiac troponin and brain natriuretic peptide in this study. However, we have to recognize that as stated in the 2016 ESC Position Paper on cancer treatments, the role of routine cardiac biomarker measurement for detecting CTRCD is not clearly established and needs further investigation^16^.

## Conclusions

It is well recognized that cancer therapy-related cardiotoxicity represents an emerging problem for cancer survivors. Nonetheless, neither conventional echocardiographic parameters nor the CTCAE heart failure score could detect subtle LV dysfunction after cancer therapy. LV GLS derived from STE is a feasible, noninvasive, and objective modality for detecting early cardiac dysfunction in lymphoma patients receiving anticancer therapy. Patients with subtle LV systolic dysfunction not only had a reduced exercise capacity, as shown by the CPET study, but also may have a higher risk of all-cause mortality and heart failure events.

## Supplementary Information

Supplementary Information
