# Supplementary material for: Subtle cardiac dysfunction in lymphoma patients receiving low to moderate dose chemotherapy
Source: Sci Rep. 2021 Mar 29;11:7100. doi: 10.1038/s41598-021-86652-x (PMC8007606; doi:10.1038/s41598-021-86652-x)
Supplement: Supplementary file 1 — Supplementary Information [file 41598_2021_86652_MOESM1_ESM.docx]

Subtle cardiac dysfunction in lymphoma patients receiving low to moderate dose chemotherapy

Hsien-Yuan Chang^1,2,^*, Chun-Hui Lee^1,3,^*, Po-Lan Su^4^, Sin-Syue Li^1,3^, Ming-Yueh Chen^3^, Ya-Ping Chen^3^, Ya-Ting Hsu^3^, Wei-Chuan Tsai^2^, Ping-Yen Liu^1,2^, Tsai-Yun Chen^3^, Yen-Wen Liu^1,2^

^1^ Institute of Clinical Medicine, College of Medicine, National Cheng Kung University, Tainan, Taiwan

^2^ Division of Cardiology, Department of Internal Medicine, National Cheng Kung University Hospital, College of Medicine, National Cheng Kung University, Tainan, Taiwan

^3^Division of Hematology and Oncology, Department of Internal Medicine, National Cheng Kung University Hospital, College of Medicine, National Cheng Kung University, Tainan, Taiwan

^4^Division of Chest, Department of Internal Medicine, National Cheng Kung University Hospital, College of Medicine, National Cheng Kung University, Tainan, Taiwan


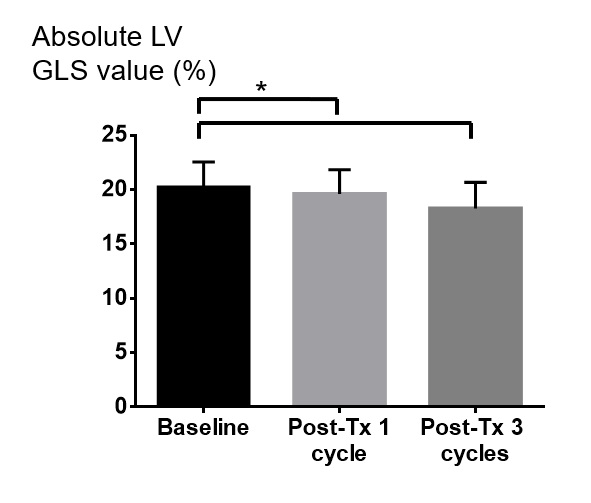


Supplemental figure 1. GLS in baseline, post treatment 1 cycle, and post treatment 3 cycles. There are 41 patients received additional GLS exam post treatment 1 cycle. Among these patients, GLS is significant decreased between baseline and post treatment 1 cycle (-20.27 ± 2.25 *vs*. -19.62 ± 2.18, *p* =0.05), and between baseline and post treatment 3 cycle (-20.27 ± 2.25 *vs*. 18.27 ± 2.38, *p*<0.001).


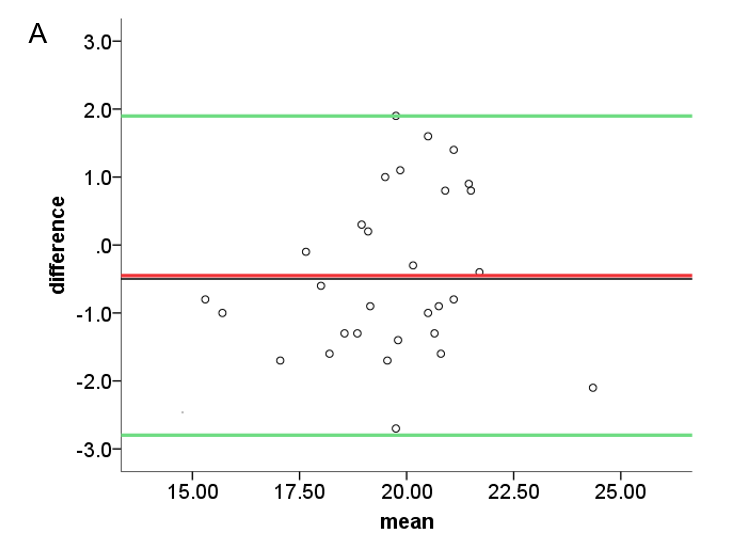

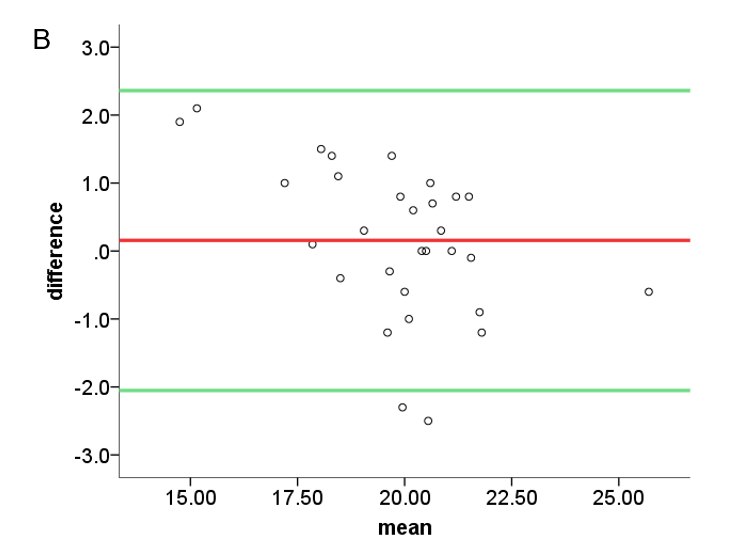


Supplemental figure 2. Bland and Altman plot for repeatability evaluation of global longitudinal strain. The solid red line showed the mean difference, and the solid green lines showed the limits of agreement by the upper and lower dotted lines and the 95%. A) difference between intra-observer. B) difference between inter-observers.

Supplemental table 1. Blood pressure and heart rate during echocardiographic measurement.

|  | Total  (N = 74) | CTRCD  (N = 36) | Non-CTRCD  (N = 38) | *p* |
| --- | --- | --- | --- | --- |
| Baseline (prior to cancer therapy) | | | | |
| SBP (mmHg) | 130 ± 15 | 129 ± 14 | 130 ± 16 | 0.9 |
| DBP (mmHg) | 75 ± 10 | 75 ± 11 | 76 ± 9 | 0.78 |
| HR (beats/minute) | 84 ± 11 | 86 ± 9 | 83 ± 12 | 0.40 |
| Post-treatment 3 cycles | | | | |
| SBP (mmHg) | 125 ± 14 | 126 ± 14 | 124 ± 14 | 0.66 |
| DBP (mmHg) | 73 ± 10 | 74 ± 11 | 72 ± 9 | 0.43 |
| HR (beats/minute) | 86 ± 13 | 85 ± 12 | 86 ± 15 | 0.95 |
| Post-treatment 6 cycles | | | | |
| SBP (mmHg) | 126 ± 13 | 125 ± 13 | 127 ± 13 | 0.67 |
| DBP (mmHg) | 75 ± 10 | 76 ± 10 | 74 ± 9 | 0.38 |
| HR (beats/minute) | 85 ± 13 | 86 ± 12 | 84 ± 14 | 0.61 |
| Post-treatment 1 year | | | | |
| SBP (mmHg) | 131 ± 20 | 132 ± 18 | 128 ± 24 | 0.67 |
| DBP (mmHg) | 77 ± 11 | 78 ± 9 | 74 ± 13 | 0.25 |
| HR (beats/minute) | 88 ± 13 | 89 ± 12 | 87 ± 15 | 0.66 |

Abbreviation. CTRCD indicates cancer therapy related cardiac dysfunction; DBP, diastolic blood pressure; HR, heart rate; SBP, systolic blood pressure.

Supplemental table 2. Independent factors in lymphoma patients with anti-cancer therapy related subtle left ventricular dysfunction

|  | Univariate | | Multivariate | |
| --- | --- | --- | --- | --- |
|  | HR (95% C.I.) | *p* | HR (95% C.I.) | *p* |
| Age (years) | 1.01 (0.98 – 1.04) | 0.41 | 1.00 (0.96 – 1.03) | 0.78 |
| Male | 3.58 (1.35 – 9.46) | 0.01 | 3.38 (1.02 – 11.1) | 0.046 |
| Hemoglobin < 11 g/dL | 3.73 (1.17 – 11.9) | 0.03 | 4.29 (1.09 – 16.9) | 0.04 |
| Doxorubicin cumulative dose | 1.00 (0.98 – 1.04) | 0.15 | 1.00 (1.00 – 1.01) | 0.33 |

Anti-cancer therapy related subtle left ventricular dysfunction was defined as a relative 15% reduction of left ventricular global peak systolic longitudinal strain from baseline (i.e. prior to anti-cancer therapy).

Abbreviation: C.I., confidence interval; HR, hazard ratio.
